# Transdifferentiation-inducing *HCCR-1 *oncogene

**DOI:** 10.1186/1471-2121-11-49

**Published:** 2010-06-30

**Authors:** Seon-Ah Ha, Hyun K Kim, JinAh Yoo, SangHee Kim, Seung M Shin, Youn S Lee, Soo Y Hur, Yong W Kim, Tae E Kim, Yeun J Chung, Shin S Jeun, Dong W Kim, Yong G Park, Jin Kim, Soon Y Shin, Young H Lee, Jin W Kim

**Affiliations:** 1 Molecular Genetic Laboratory, Catholic Medical Research Institute, The Catholic University of Korea, Seoul, Korea; 2 Department of Clinical Pathology, College of Medicine, The Catholic University of Korea, Seoul, Korea; 3 Department of Obstetrics and Gynecology, College of Medicine, The Catholic University of Korea, Seoul, Korea; 4 Department of Microbiology, College of Medicine, The Catholic University of Korea, Seoul, Korea; 5 Department of Neurosurgery, College of Medicine, The Catholic University of Korea, Seoul, Korea; 6 Department of Internal Medicine, College of Medicine, The Catholic University of Korea, Seoul, Korea; 7 Department of Biostatistics, College of Medicine, The Catholic University of Korea, Seoul, Korea; 8 Department of Anatomy, College of Medicine, The Catholic University of Korea, Seoul, Korea; 9 Department of Biomedical Science and Technology, Research Center for Transcription Control, Institute of Biomedical Science and Technology, Konkuk University, Seoul, Korea

## Abstract

**Background:**

Cell transdifferentiation is characterized by loss of some phenotypes along with acquisition of new phenotypes in differentiated cells. The differentiated state of a given cell is not irreversible. It depends on the up- and downregulation exerted by specific molecules.

**Results:**

We report here that *HCCR-1*, previously shown to play an oncogenic role in human cancers, induces epithelial-to-mesenchymal transition (EMT) and mesenchymal-to-epithelial transition (MET) in human and mouse, respectively. The stem cell factor receptor CD117/c-Kit was induced in this transdifferentiated (EMT) sarcoma tissues. This MET occurring in *HCCR-1 *transfected cells is reminiscent of the transdifferentiation process during nephrogenesis. Indeed, expression of *HCCR-1 *was observed during the embryonic development of the kidney. This suggests that *HCCR-1 *might be involved in the transdifferentiation process of cancer stem cell.

**Conclusions:**

Therefore, we propose that *HCCR-1 *may be a regulatory factor that stimulates morphogenesis of epithelia or mesenchyme during neoplastic transformation.

## Background

The concept that genetic events cooperate to achieve malignant transformation was proposed over a decade ago. Primary rodent cells are efficiently converted into tumorigenic cells by the co-expression of cooperating oncogenes. However, similar experiments with human cells have consistently failed [1]. In 1999, after more than 15 years of trying, researchers have managed to convert normal human cells into tumor cells by delivering telomerase catalytic subunit in combination with two oncogenes [2]. Although malignant transformation of human cells by a single oncogene may not occur or may require specialized factors, we demonstrated that *HCCR-1*, associated with various types of human cancers, alone induced tumorigenic conversion of mouse cells [3].

We have identified a novel oncogene, human cervical cancer oncogene (*HCCR*), that was classified into 2 types: *HCCR-1 *(GenBank accession number AF 195651) and *HCCR-2 *(GenBank accession number AF 315598) [3]. The *HCCR-1 *and *HCCR-2 *overexpressed cells were tumorigenic in nude mice and *HCCR *transgenic mice developed breast cancers and metastasis [3,4]. Also, *HCCR-1 *was overexpressed in various types of human malignancies and was found to regulate the p53 tumor-suppressor gene negatively [3-6]. However, it is unknown how *HCCR-1 *contributes to the cellular and biochemical mechanisms of human tumorigenesis.

Cell transdifferentiation is characterized by loss of some phenotypesalong with acquisition of new phenotypes in differentiated cells. Differentiated cells are endowed with the capacity of transforming into cells of a different type having other functions [7]. Gene expression in differentiated cells has long been considered an irreversible phenomenon that is established at the time of replication. Given that, although repressed, the same genetic framework is present in all cell types, a change in gene expression among differentiated cells was predictable in particular conditions.In fact, the differentiated state of a given cell is not irreversible.It depends on the up- and downregulation exerted by specificmolecules [8].

Recent research suggests that tumor formation may result from the development of cancer stem cells by the deregulation of normal self-renewal pathways of tissue stem cells. Numerous signalling pathways have been implicated in this process including Notch, Wnt, LIF (leukemia inhibitory factor), PTEN (phosphatase and tensin homologue deleted from chromosome 10), SHH (sonic hedgehog) and BMI1 [9-12]. The discovery of cancer stem cells in AML, breast cancer and some CNS tumors offers a new approach to understanding the biology of these conditions. Further study into these and other mechanisms controlling self-renewal pathways is needed to understand not only what drives tumor formation from cancer stem cells but also what mechanisms could be used to 'switch off' tumor formation [13].

We undertook this study with the following aims: 1) to assess whether *HCCR-1 *overexpression converts normal cells to malignant transformed cells; 2) to determine whether *HCCR-1 *is involved in transdifferentiation process and embryonic kidney development; 3) to examine the molecular alterations occurring in *HCCR-1 *induced tumorigenesis.

## Results

### HCCR-1 is involved in tumorigenesis and transdifferentiation

We investigated whether *HCCR-1 *alone can induce malignant transformation of HEK-293 cells. Transfection of *HCCR-1 *expression vector into HEK-293 cells yielded a transformation efficiency (*i.e*., the number of foci per μg of DNA transfected) of 0.9 (Table 1). The transformation efficiency of the wild-type *ras *gene used as a positive control was 1.2 (95% CI, 16.9-19.1) (Table 1). These data suggest that *HCCR-1 *has almost as potent oncogenic activity as wild-type *ras *in the HEK-293 assay system. Comparable transfection with the control vector alone (nontransformed 293 cells) yielded no transformation (Table 1). In total, 20 of the transformed foci were isolated from the *HCCR-1 *transfected 293 cells and were grown in semisolid soft-agar medium to measure the colony formation efficiency. *HCCR-1*-transfected 293 cells formed colonies with an efficiency of 13%.

**Table 1:** Transforming activity of *HCCR-1 *in HEK-293 cells

Transfected DNA source	Foci/μg DNA*	Transformation efficiency**	95% confidence intervals
HCCR-1	13/15	0.9	11.2-13.8
Wild-type ras	18/15	1.2	16.0-19.1
Vector alone	0/15	0	-

*HEK-293cells (3 × 10^5 ^cells) were transfected with 5 μg of DNA obtained from indicated source. The results reported here were derived from 3 independent experiments.

**Transformed cell aggregates, foci, were counted by visual examination starting 2-3 weeks after transfection and the transformation efficiencies (*i.e*., the number of foci per microgram of DNA transfected) were determined.

**Figure 1:**
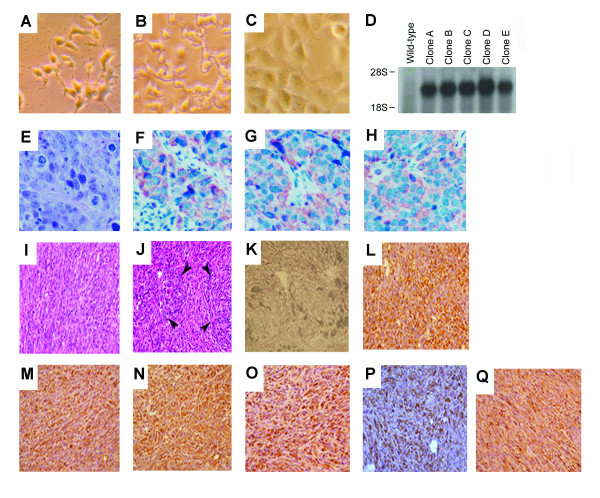
***HCCR-1 *is ivolved in tumorigenic conversion and transdifferentiation**. Phase-contrast features of wild-type 293 cells **(A)**, cells transfected vector alone **(B) **and *HCCR-1*-transfected human 293 cells **(C)**. **D**. *HCCR-1 *mRNA expressions in *HCCR-1*-transfected human 293 cells (designated as clone A, B, C, D, and E, consecutively), and wild-type human 293 cells. **E**. Hematoxylin-eosin (H-E) staining of tumor nodules taken from the nude mice xenograft. The tumor cells show similar histological features with *HCCR-1*-transfected allografts. Tumor cells are arranged in nests separated by fine fibrovascular septae and have polygonal shape with oval nuclei, coarse chromatin and moderate amount of cytoplasm. Numerous mitotic figures are found. Immunohistochemical stainings of *HCCR-1*-transfected human 293 cells xenograft. Tumor cells show positive cytoplasmic stainings for cytokeratin 8 **(F)**, cytokeratin 19 **(G)**, and vimentin **(H)**, respectively. Magnifications, **E**) × 300; **F**-**H**) × 250. Tumorigenic conversion and transdifferentiation of NIH/3T3 cells after transfection with *HCCR-1*. **I and J**. H-E staining of tumor nodules taken from nude mice after 3 weeks injection with *HCCR-1*-transfected NIH/3T3 cells. [**I**, tumor sections show malignant spindle cell sarcoma; **J**, tumor sections show poorly differentiated sarcoma with focal epithelial differentiation (arrows)]. Magnification × 400. **K**. Reticulin stain was performed to detect reticulin fibers. Sections from the same tumor nodule were immunohistochemically stained with mouse monoclonal antibodies (DAKO) specific for epithelial membrane antigen **(L)**, cytokeratin 7 **(M)**, cytokeratin 8 **(N)**, cytokeratin 19 **(O)**, cytokeratin 20 **(P)**, and vimentin **(Q)**. DAB was used as the chromogen. After immunostaining, sections were counterstained with hematoxylin. Magnification × 200.

HEK-293 is a short spindle shaped cell having a bipolar cell process (Figure 1A). Cultured 293 cells have similar cytological features to cells transfected with vector alone (Figure 1B). However, *HCCR-1*-transfected human 293 cells showed increase in cell size compared to wild-type human 293 cells (Figure 1C). The cell processes were blunted in *HCCR-1*-transfected human 293 cells. Northern blot showed that about 2.0-kilobase-pair mRNA transcript was over-expressed in all five *HCCR-1*-transfected human 293 cells as compared with wild-type 293 cells (Figure 1D). Nude mice injected with *HCCR-1*-transfected human 293 cells showed palpable tumors in four weeks. But cells transfected with vector alone did not induce tumor formation in nude mice. Sections of the nude mice tumor nodules bearing *HCCR-1*-transfected human 293 cells revealed epithelial cell carcinomas (Figure 1E). Interestingly, however, the cells showed co-expression of epithelial markers, such as cytokeratin 8 (Figure 1F) and cytokeratin 19 (Figure 1G), and of the mesenchymal marker, vimentin that is normally expressed by fibroblasts (Figure 1H). These results suggest that transdifferentiation (EMT) occurred in *HCCR-1 *stably transfected 293 cells derived from nude mouse tumors.

In order to confirm the above data, we performed the immunofluorescence microscopy experiment in HEK-293 and HEK-293 stable clone for *HCCR-1 *(Additional file 1 Figure S1 A, B). The result showed that the parental HEK-293 cells express the vimentin but in a lower level than *HCCR-1 *stable HEK-293 clone. This suggests that the vimentin level is slightly increased after *HCCR-1 *transfection. We then analyzed other epithelial markers in HEK-293, HEK-293 clones stably transfected with *HCCR-1*, and HEK-293 cells transfected with the empty vector. E-cadherin, α-catenin, and β-catenin are essential for the maintenance of epithelial structures. To determine whether the over-expression of *HCCR-1 *alters the expression profiles of these molecules, we performed the western blotting analyses as follows (Additional file 2 Figure S1 C). The result demonstrates that all of these epithelial markers (E-cadherin, α-catenin, and β-catenin) are down-regulated only in *HCCR-1 *stable cell lines. Therefore, this data support that *HCCR-1 *induces EMT in transformed HEK-293 cells.

The oncogenic transformation of NIH/3T3 cells would typically be expected to develop sarcomas because NIH/3T3 cells are of mesenchymal origin [14]. However, the tumors in nude mice derived from *HCCR-1 *stably transfected NIH/3T3 cells had both sarcomatous (Figure 1I) and epithelial features (Figure 1J). Histologically, the tumors showed poorly differentiated sarcoma with epithelial differentiation (carcinosarcoma) (Figure 1J). They were composed mostly of spindle cells forming long and short fasciles. In focal areas these cells were vaguely aggregated to form epithelial cell nest-like structures (arrows). Reticulin fibers enveloped individual spindle cells in the sarcomatous areas, but enveloped vague epithelial cell nests in the more carcinomatous areas (Figure 1K). For a morphologic comparison between *HCCR-1*-derived tumor cells and epithelial cells, we determined whether *HCCR-1*-derived tumor cells expressed epithelial cell markers, such as the epithelial membrane antigens (Figure 1L), cytokeratin 7 (Figure 1M), cytokeratin 8 (Figure 1N), cytokeratin 19 (Figure 1O), cytokeratin 20 (Figure 1P), and the mesenchymal marker vimentin (Figure 1Q). *HCCR-1*-derived tumor cells were positive for both epithelial and mesenchymal markers (Figures 1L-1Q). These results suggest that transdifferentiation (MET) occurred in *HCCR-1 *stably transfected NIH/3T3 cells derived from nude mice tumors. *HCCR-1 *might play multiple developmental roles by mediating a signal originating from the mesenchyme and received by epithelia. Mesenchymal signals are known to govern differentiation and morphogenesis of many epithelia, but the molecular nature of the signals is poorly understood. This expression pattern indicates that this mesenchymal factor can transmit morphogenetic signals in epithelia development and suggests a molecular mechanisim for mesenchymal epithelial interactions. This study indicates that the *HCCR-1 *oncogene may be a mesenchyme-derived cytokine that stimulates the morphogenesis of epithelia and mediates interactions between the mesenchyme and epithelia during neoplasia.

### Induction of c-kit proto-oncogene product by HCCR-1

**Figure 2:**
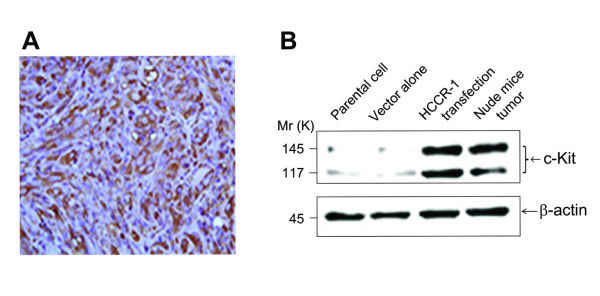
***HCCR-1 *induced the *c-kit *proto-oncogene product**. *HCCR-1 *induced the *c-kit *proto-oncogene product. **A**. Sections were stained for CD117. **B**. Western blot analysis to determine the expression of c-kit proto-oncogene product. *HCCR-1 *gene in embryonic kidney development.

CD117 (c-Kit) is a type III receptor tyrosine kinase operating in cell signal transduction in several cell types. Normally c-Kit is activated (phosphorylated) by binding of its ligand, the stem cell factor (SCF) [15]. This leads to a phosphorylation cascade ultimately activating various transcription factors in different cell types. Such activation regulates apoptosis, cell differentiation, proliferation, chemotaxis, and cell adhesion. Ligand independent activation of c-Kit (dysregulated kit function) has been found to be an important component of oncogenesis in a large number of neoplastic disorders such as systemic mastocytosis, germ cell tumors, acute myelogenous leukemia (AML) with the disruption of the core binding factor, amongst others [16]. C-Kit positivity has been variably reported in sarcomas [16]. Among mesenchymal tumors, c-kit seems to be specific for the gastrointestinal stromal tumors (GISTs), which consistently express this protein. These tumors uniformly express CD117, the *c-Kit *proto-oncogene product [15]. Signal transductions from tyrosine kinase receptors have key roles in the regulation of cellular proliferation and differentiation [17] and binding of SCF to c-Kit activates multiple signal transduction pathways, including phosphatidylinositol 3-kinase (PI3K)/Akt [18]. Because *HCCR-1 *induced carcinosarcoma and *HCCR-1 *is regulated by the PI3K/Akt signaling pathway [19], we investigated whether *HCCR-1*-induced nude mice-derived carcinosarcoma induces the expression of CD117, as does GIST. *HCCR-1*-derived tumor cells showed a positive staining for CD117 (Figure 2A). Western blot analysis also showed that NIH/3T3 cells stably transfected with *HCCR-1 *and nude mice-derived tumors injected with NIH/3T3 cells transfected with *HCCR-1 *both overexpressed the c-kit protein compared with NIH/3T3 parental cells or cells transfected with vector alone (Figure 2B). Our study shows that *HCCR-1*-derived tumor cells express CD117, suggesting that *HCCR-1 *is related to the c-kit signaling pathway.

### Embryonic kidney development

Co-expression of the human Met receptor, its ligand, and hepatocyte growth factor/scatter factor (HGF/SF), in NIH/3T3 fibroblasts causes cells to become tumorigenic in nude mice. The resultant tumors display lumen-like morphology, contain carcinoma-like focal areas with intercellular junctions resembling desmosomes, and co-express epithelial (cytokeratin) and mesenchymal (vimentin) cytoskeletal markers. The apparent MET of the tumor cells mimics the conversion that occurs during embryonic kidney development, suggesting that Met-HGF/SF signaling plays a role in this process as well as in tumors that express both epithelial and mesenchymal markers [20]. Because acquisition of epithelial properties by the fibroblast-derived cells mimics the MET of cells during the organogenesis of the kidney [20], we investigated whether *HCCR-1 *is expressed in the developing kidney. Immunoblot analysis demonstrated that as probed by rabbit polyclonal anti-HCCR-1 serum, HCCR-1 began to be overexpressed at fetal 18-day, remaining high up to postnatal 14-day, and decreased to a very low level in adult rat kidney (Additional file 2 Figure S2 A). Sections of 20-day-old fetal rat kidney revealed that *HCCR-1 *antibody stained throughout the collecting ducts only (Additional file 1 Figure S2 B, medulla on the left side), which are derived from the ureteric bud [21]. The developing nephrons in the cortex were not stained (Additional file 1 Figure S2 B, nephrogenic zone on the right side). But the basolateral plasma membranes of the developing collecting duct, which are derived from the ureteric bud, were especially reactive to HCCR-1 antibody (Additional file 1 Figure S2 C). Because nephrogenesis is stimulated by a distinct ureteric signal, diffusion-limited basolateral molecules [22], which trigger MET, we propose that the *HCCR-1 *product may be a mesenchyme-derived regulatory factor [23]that stimulates morphogenesis of epithelia in the developmental process and mediates interactions between mesenchyme and epithelia during neoplastic transformation.

### Molecular genetic alterations in the HCCR-1-induced tumorigenesis

**Figure 3:**
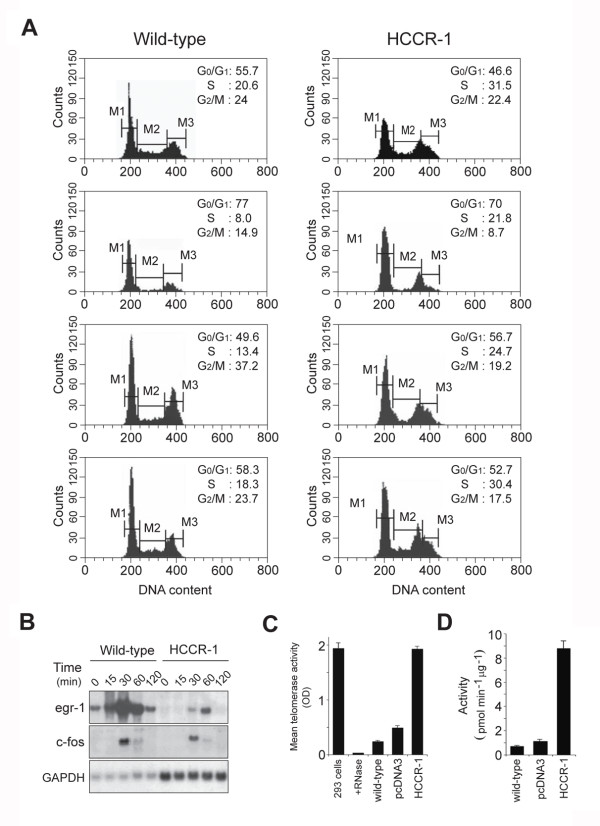
**Molecular alterations in *HCCR-1*-induced tumorigenesis**. **A**. Cell-cycle profiles of wild-type NIH/3T3 cell and *HCCR-1*-transfected cells from a separate experiment. Exponentially growing cells were trypsinized and DNA content was determined by flow cytometry. To assess the serum-dependent cell cycle progression, cells were cultured in 0.5% BCS for 36 houes. After incubation, cells were released with 20% serum and harvested. **B**. Tumor suppressor egr-1 expressions. *HCCR-1*-transfected cells were serum starved with 0.5% BCS for 36 hours, and then stimulated with fresh 20% serum. Total RNA was isolated, transferred, and hybridized with ^32^P-labeled egr-1, c-fos, and GAPDH probe, respectively. **C**. Determination of telomerase activity. Human telomerase-positive embryonic kidney 293 cells, 293 cell extracts treated with RNase (+ RNase), wild-type NIH/3T3, cells transfected with vector alone and *HCCR-1*-transfected cells were analyzed by Telomerase PCR ELISA. Assays were performed according to the kit protocol with amounts of extracts equivalent to 1 ×10 ^3 ^cells. The telomerase activity in 293 cells, which served as a positive control, was abolished by pretreatment with RNase. Results are the average mean absorbance values from four separate experiments (means and 95% confidence intervals) (wild-type versus *HCCR-1*, *P *< .05). **D**. Determination of PKC activity. Wild-type NIH/3T3, cells transfected with vector alone and *HCCR-1*-transfected cell extracts were prepared and assayed to determine PKC activity. Each value is the means and 95% confidence intervals of three independent experiments (wild-type versus *HCCR-1*, *P *< 0.05).

In order to study whether there was an alteration in the growth properties of *HCCR-1*-transfected cells, we examined cell cycle profiles. The percentage of wild-type NIH/3T3 cells and *HCCR-1*-transfected cells in the S-phase was 20.6% and 31.5%, respectively (Figure 3A, mid-log phase). These results suggest that there was a significant shift of the cell population out of the G _0_/G _1_-phase into the S-phase in *HCCR-1*-transfected cells. To assess the serum-dependent cell cycle progression, cells were cultured in 0.5% bovine calf serum (BCS) for 36 hours. After incubation, cells were released with 20% serum and harvested at the indicated times. In wild-type cells (measured at 0 hour), few cells remained in the S-phase (8%). In contrast, a considerable number of *HCCR-1*-transfected cells were still in the S-phase (21.8%), suggesting that constitutive overexpression of *HCCR-1 *allowed for a relative amount of resistance to serum deprivation-induced G _0_/G _1 _arrest. Following the release of cells from the growth arrest caused by serum-deprivation, there were consistent increases of over 10% in the S-phase populations of *HCCR-1*-transfected cells as compared to wild-type cells at measured time intervals (24 hour and 48 hour, respectively). Therefore, overexpression of *HCCR-1 *could deregulate cell growth by shortening the G _0_/G _1_-phase and increasing the S-phase population of cells. It has been implicated that egr-1 functions as a tumor-suppressor [24]. To assess whether *HCCR-1*-induced tumor formation is associated with the loss of egr-1 expression, we tested the time course of egr-1 expression. When quiescent cells were stimulated with 20% serum, a marked down-regulation of egr-1 was observed in *HCCR-1*-transfected cells compared with wild-type NIH/3T3 cells (Figure 3B). In contrast, upregulation of GAPDH mRNA level in *HCCR-1*-transfected cells was clearly seen, while no significant difference was observed in the level of c-fos (Figure 3B). This result suggests that down-regulation of tumor suppressor egr-1 may be involved in the tumor progression in *HCCR-1*-overexpressing cells. To further explain the tumorigenesis of *HCCR-1*, we determined the telomerase activity in PKC-activated *HCCR-1*-transfected cells. Consistent with a previous study [25],wild-type NIH/3T3 cells showed detectable telomerase activity (Figure 3C). However, *HCCR-1 *gene transfection increased telomerase activity up to about 7-fold when compared with wild-type cells. Reports show that PKC induces a marked increase in telomerase activity [26]. To determine whether the increased telomerase activity in *HCCR-1 *transfected cells is caused by PKC, a kinase assay was performed. PKC activity of *HCCR-1*-transfected cells was increased by about 10-fold when compared with wild-type cells (Figure 3D).

## Discussion

The conversion of normal cells into tumor cells involves changes in the activity of a number of distinct different genes and proteins in a cell. Although researchers have been able to transform normal mouse cells into tumor-forming cells by introducing several cooperating oncogenes into these cells, human cells have been resistant to such transformation [8,27-29]. In this study, ectopic expression of *HCCR-1 *alone results in direct tumorigenic conversion of HEK-293 cells *in vitro *and *in vivo*.

Because NIH/3T3 cell is of mesenchymal origin [14],sarcoma would typically develop from oncogene-transformed NIH/3T3 [30]. But, nude mice bearing *HCCR-1 *allograft display characteristics of epithelial carcinomas. Because acquisition of epithelial properties by the fibroblast-derived cells mimics the mesenchymal to epithelial conversion of cells during the organogenesis of the kidney [20], we investigated whether *HCCR-1 *is expressed in the developing kidney. The developing nephrons in the cortex were not stained. But the basolateral plasma membranes of the developing collecting duct, which are derived from the ureteric bud [21],were especially reactive to HCCR-1 antibody. Because nephrogenesis is stimulated by a distinct ureteric signal, diffusion-limited basolateral molecules [22],which trigger mesenchymal to epithelial conversion, we propose that the *HCCR-1 *product may be a mesenchyme-derived regulatory factor [23]that stimulates morphogenesis of epithelia in the developmental process and mediates interactions between mesenchyme and epithelia during neoplastic transformation. Our study suggests that overexpression of *HCCR-1 *induces tumorigenesis, transdifferentiation and embryonic kidney development.

Transdifferentiation is a change from one differentiated phenotype to another involving morphological and functional phenotypic markers [31,32]. The conversion of a cell phenotype is likely to be accomplished by selective enhancement of gene expression, which controls the terminal developmental commitment of cells [33].There is little known about 'master switch' genes that determine a specific differentiation pathway and have the potential to induce the process in a cell originally destined for a different differentiation pathway [31].*HCCR-1 *might play multiple developmental roles by mediating a signal originating from the mesenchyme and received by epithelia. Mesenchymal signals are known to govern differentiation and morphogenesis of many epithelia, but the molecular nature of the signals is poorly understood. This expression pattern indicates that this mesenchymal factor can transmit morphogenetic signals in epithelia development and suggests a molecular mechanisim for mesenchymal epithelial interactions.

There is evidence to indicate that tumors develop secondarily to abnormalities in PKC-mediated signal transduction [34].Reports show that PKC induces a marked increase in telomerase activity [26].Besides tumor cells typically have acquired damage to genes that directly regulate their cell cycles [35]. Our study suggests that deregulation of *HCCR-1 *activity in mouse NIH/3T3 cells might result in the activation of PKC or telomerase, loss of particular cell cycle checkpoint controls, and downregulation of tumor suppressor egr-1, thereby predisposing NIH/3T3 cells to malignant conversion.

This present study suggests that *HCCR-1 *is an oncogene which induces the transformation of HEK293 and NIH3T3 cells. Likewise, our previous study also demonstrated that *HCCR-1 *is a mitochondrial out membrane protein and suppresses the apoptosis [36]. Consistent with this previous work, this study also reveals the anti-apoptotic activity of *HCCR-1 *by reducing the expression of Egr-1, a direct regulator of multiple tumor suppressors including TGF beta1, PTEN, and p53. Therefore, both studies support that *HCCR-1 *is an oncogene either by suppressing apoptotic activities or by dysregulating Egr-1, telomerase, or PKC activity. Since key functions related to apoptosis or anti-apoptosis often occur in mitochondria, it is not too surprising that *HCCR-1 *localizes to the mitochondria.

## Conclusions

In conclusion, we converted normal cells into tumor cells by delivering *HCCR-1 *alone in combination with no other oncogenes. EMT and MET occurred in *HCCR-1*-transfected tumor cells. In addition, *HCCR-1 *participates in induction of the c-kit proto-oncogene, in activation of PKC and telomerase activities, and cell cycle progression. While further studies are needed to characterize cellular functions and regulatory mechanisms, *HCCR-1 *protein is likely to be a candidate onco-developmental protein for cancer stem cell in the development of human cancer.

## Methods

### Cell lines, construction of expression vector, and DNA transfection

Human embryonic kidney (HEK) 293 (ATCC CRL-1573) and NIH/3T3 cells were obtained from the ATCC. HEK-293/HCCR-1-V5 cell lineswere maintained in DMEM (Gibco) containing 200 μg/ml G418, 10% FBS and 1% PenStrep (Gibco).

Expression vector containing the coding region of *HCCR-1 *was constructed as follows. First, the *Sal*I fragment was isolated from the prokaryotic expression vector, pCEV-LAC, which contains the entire *HCCR-1 *cDNA. Then, pcDNA3.1 (Invitrogen, CA) was digested with *Xho*I to make a compatible end with *Sal*I. A *Sal*I fragment containing the *HCCR-1 *coding sequence was inserted into the *Xho*I-digested pcDNA3.1. Lipofectamine (Gibco BRL, Rockville, MD) was used to introduce the *HCCR-1 *expression vector into HEK-293 cells.

### Morphology and tumorigenecity

Newly established cells grown in culture flasks were photographed by phase-contrast microscopy. To analyze tumorigenecity, 5 ×10 ^6 ^cells were injected subcutaneously into the posterior lateral aspect of the trunk of mice (5-week-old athymic nu/nu on BALB/c background). Nude mice were sacrificed when the subcutaneous tumors reached 1.5-2.5 cm in diameter.

### Colony-forming efficiency

Five ×10 ^3 ^viable cells were suspended in 1 ml of 0.3% noble agar (Difco Laboratories Inc., Detroit, MI) made with complete media, and layered onto 0.6% agar in 35-mm plates. All samples were plated in quadruplicate. The number of cell colonies (>50 cells/cluster) was estimated on days 21-28.

### Immunoblot analysis and immunohistochemistry

For immunoblot analysis, cells were lysed in Laemmli sample buffer. Proteins were separated by 10% SDS-PAGE and then electroblotted. The membranes were incubated with a rabbit polyclonal anti-HCCR-1 serum and proteins were revealed by an ECL-Western blot detection kit. We performed immunohistochemistry on cryosections (5-μm) incubated with anti-vimentin, anti-keratin, anti-EMA antibodies (DAKO), and polyclonal antibody raised against HCCR-1. Binding of primary antibody was visualized by biotinylated secondary antibody, avidin-horseradish peroxidase, and AEC as the chromogen.

### PKC and telomerase activity assays

PKC activity was measured using the SignaTECT™ Protein Kinase C Assay System (Promega, Madison, WI). PKC activity was defined as the difference in counts per minute incorporated into substrate in the absence and presence of phospholipid. Telomerase activity was determined by using the telomerase PCR-ELISA kit (Boehringer Mannheim). Immortalized human kidney cells (293 cells) provided in the kit were used as the positive control. A negative control was provided for human 293 cells by pretreatment with RNase.

### Cell cycle experiments

Cells cultured at mid-log phase were growth arrested by incubation in medium containing 0.5% bovine calf serum for 36 hours. Cells to be analyzed for DNA content were harvested following trypsinization, and fixed in 70% ethanol. Fixed cells were then stained with propidium iodide. In brief, 50 μg/ml of propidium iodide staining solution (Sigma) and 100 units per ml of RNase A (Boerhinger Mannheim) were added to 2 ×10 ^6 ^cells. After incubation for 1 hour, cellular DNA content was determined by fluorescence analysis at 488 nm using a FACS Caliber (Becton Dickinson). A minimum of 1 ×10 ^4 ^cells per sample was analyzed with Modfit 5.2 software.

### Statistical analysis

Data was analyzed by use of SAS software (SAS Institute, Cary, NC). One-way analysis of variance was used for comparing various outcome measures (e.g., PKC or telomerase levels) in different experimental conditions. The mean values and 95% confidence intervals for the outcome variables are shown in relevant figures. All reported *P *values are two-sided and were considered to be statistically significant at the .05 level. *P *values for comparing the difference between groups are adjusted by Dunnett's multiple comparisons.

## Authors' contributions

SH, HKK, JY, SK and SMS performed and designed experiments, analyzed data and assisted in writing the manuscript. YGP analyzed bioinformatics data, and YSL conducted histological analysis. YWK, TEK, YJC, SSJ, DWK, JK (from the Catholic) and SYS, YHL (from the Konkuk) were collaborators on the paper. JWK designed experiments, interpreted and assisted in writing the manuscript. SH and HKK contributed equally to this work. All authors read and approved the manuscript.

## Supplementary Material

Additional file 1 **Expression analysis of vimentin**. (**A, B**) by immunofluorescence and epithelial markers such as E-cadherin, α-catenin, and β-catenin (**C**) by western blotting experiments. HEK-293 parental cells (**A**) and HEK-293 stable clones for *HCCR-1 *(**B**) were stained for anti-vimentin antibodies. In **C**, HEK-293 parental cells (lanes 1 and 2), HEK-293 stable cells for *HCCR-1 *(lanes 3 and 4), and HEK-293 cells transfected with empty vectors (lanes 5 and 6) were analyzed by antibodies against E-cadherin, α-catenin, and β-catenin. Click here for file

Additional file 2 ***HCCR-1 *gene in embryonic kidney development**. **A**. Detection of *HCCR-1 *protein in fetal 16-, 18-, 20-, postnatal 1-, 7-, 14-day and adult rat kidney tissue extracts. Total proteins were subjected to SDS-PAGE. *HCCR-1 *positive bands were revealed by ECL-Western blot detection kit. *F *and *P *denote fetal and postnatal, respectively. **B**. Immunohistochemical staining of 20-day-old fetal rat kidney. Immunostaining was confined to the collecting ducts. Magnification, × 42. **C**. Differential-interference contrast microscopy of 20-day-old fetal rat kidney illustrating *HCCR-1 *immunostaining in the basolateral plasma membrane of medullary collecting duct. Magnification, **× **220. Click here for file
