# Supplementary figures and images for: Transdifferentiation-inducing HCCR-1 oncogene
Source: BMC Cell Biol. 2010 Jun 30;11:49. doi: 10.1186/1471-2121-11-49 (PMC2909153; doi:10.1186/1471-2121-11-49)

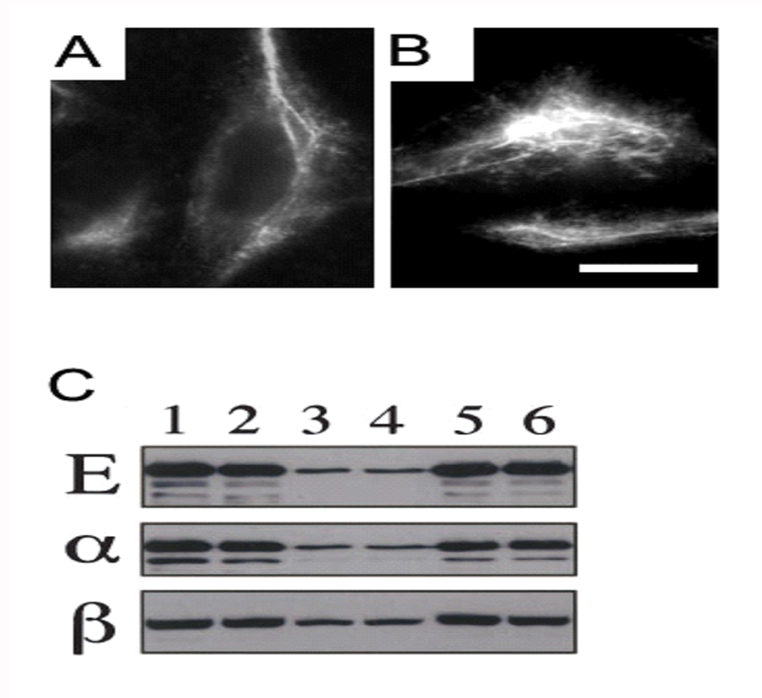

Supplement: Additional file 1 — Expression analysis of vimentin. (A, B) by immunofluorescence and epithelial markers such as E-cadherin, α-catenin, and β-catenin (C) by western blotting experiments. HEK-293 parental cells (A) and HEK-293 stable clones for HCCR-1 (B) were stained for anti-vimentin antibodies. In C, HEK-293 parental cells (lanes 1 and 2), HEK-293 stable cells for HCCR-1 (lanes 3 and 4), and HEK-293 cells transfected with empty vectors (lanes 5 and 6) were analyzed by antibodies against E-cadherin, α-catenin, and β-catenin. [file 1471-2121-11-49-S1.TIFF]

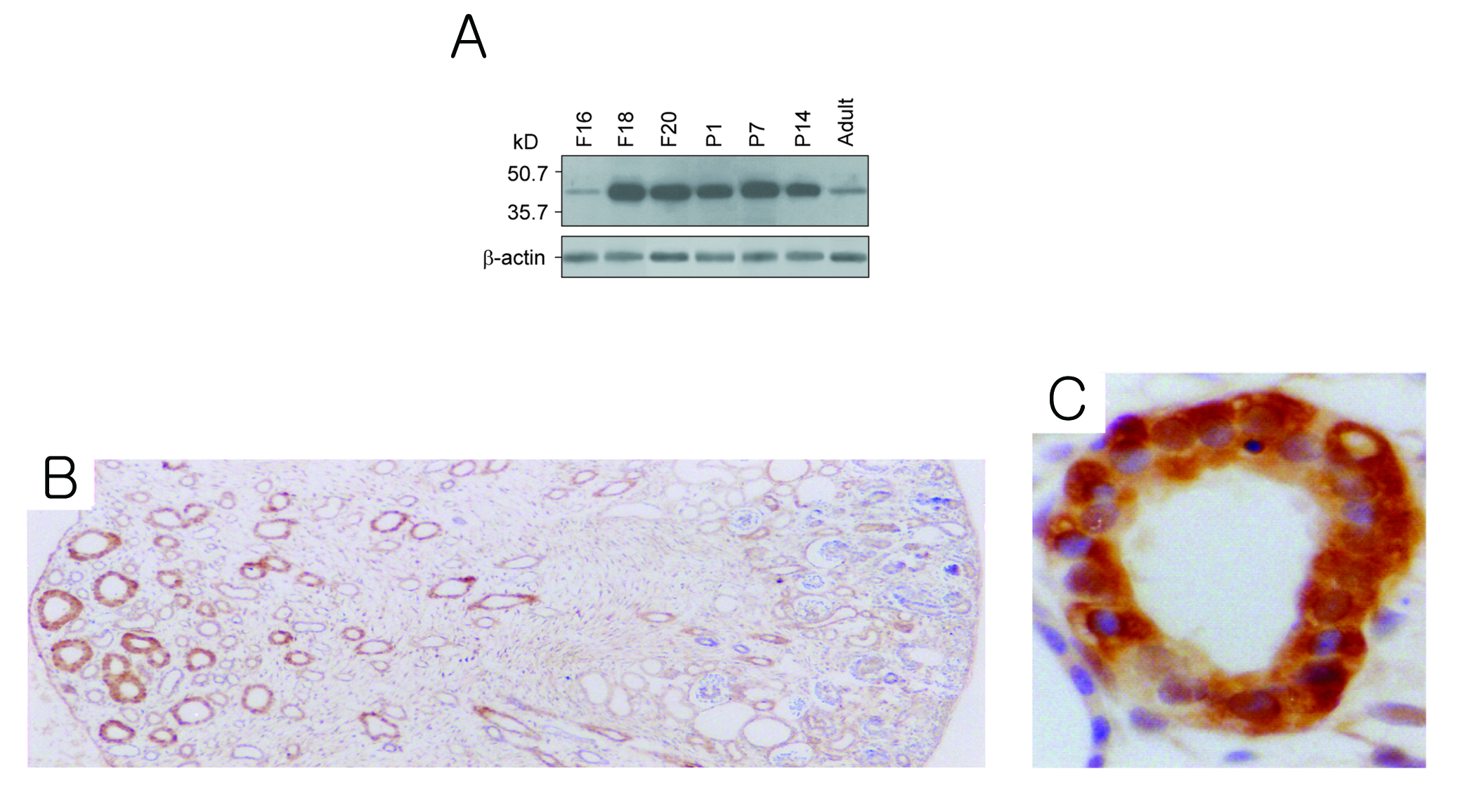

Supplement: Additional file 2 — HCCR-1 gene in embryonic kidney development. A. Detection of HCCR-1 protein in fetal 16-, 18-, 20-, postnatal 1-, 7-, 14-day and adult rat kidney tissue extracts. Total proteins were subjected to SDS-PAGE. HCCR-1 positive bands were revealed by ECL-Western blot detection kit. F and P denote fetal and postnatal, respectively. B. Immunohistochemical staining of 20-day-old fetal rat kidney. Immunostaining was confined to the collecting ducts. Magnification, × 42. C. Differential-interference contrast microscopy of 20-day-old fetal rat kidney illustrating HCCR-1 immunostaining in the basolateral plasma membrane of medullary collecting duct. Magnification, × 220. [file 1471-2121-11-49-S2.TIFF]
